# Antihyperlipidemic and Antioxidant Effects of *Averrhoa* *Carambola* Extract in High-Fat Diet-Fed Rats

**DOI:** 10.3390/biomedicines7030072

**Published:** 2019-09-16

**Authors:** Saleem H. Aladaileh, Sultan A. M. Saghir, Kisantini Murugesu, Amirin Sadikun, Ashfaq Ahmad, Gurjeet Kaur, Ayman M. Mahmoud, Vikneswaran Murugaiyah

**Affiliations:** 1 Department of Medical Analysis, Princess Aisha Bint Al-Hussein College of Nursing and Medical Sciences, Al-Hussein Bin Talal University, Ma`an 71111, Jordan; Sal_adaileh@hotmail.com; 2 Department of Pharmacology, School of Pharmaceutical Sciences, Universiti Sains Malaysia, Penang 11800, Malaysia; kishamuru@gmail.com; 3 Department of Pharmaceutical Chemistry, School of Pharmaceutical Sciences, Universiti Sains Malaysia, Penang 11800, Malaysia; amirin@usm.my; 4 Department of Pharmacology and Toxicology, Virginia CommonWealth University, Richmond, VA 23284, USA; aahmad2@vcu.edu; 5 Institute for Research in Molecular Medicine (INFORMM), Universiti Sains Malaysia, Pinang 11800, Malaysia; gurjeet@usm.my; 6 Physiology Division, Department of Zoology, Faculty of Science, Beni-Suef University, Beni-Suef 62514, Egypt; ayman.mahmoud@science.bsu.edu.eg

**Keywords:** *Averrhoacarambola*, dyslipidemia, high-fat diet, HMG-CoA, oxidative stress

## Abstract

The present study explored the antihyperlipidemic potential of a standardized methanolic extract of *Averrhoa carambola* (*A. carambola*) leaf (MEACL) in high-fat diet (HFD)-fed rats. The standardized MEACL was orally administered at different doses (250, 500, and 1000 mg/kg) to HFD-induced hyperlipidemic rats for five weeks. Serum lipid profile, body weight changes, body mass index (BMI), daily food intake, relative organ weight, and histology of the liver were evaluated. In addition, the effect of MEACL on HMG-CoA reductase and pancreatic lipase activities as well as hepatic and fecal lipids was demonstrated. MEACL supplementation reduced serum lipids in HFD-fed rats in a dose-dependent manner. Histopathological scores revealed that 1000 mg/kg MEACL restored the damage to liver tissue in hyperlipidemic rats. MEACL decreased the body mass index (BMI), atherogenic index, and hepatic cholesterol and triglycerides and increased fecal cholesterol and bile acids in HFD-fed rats. Also, MEACL ameliorated lipid peroxidation and improved antioxidant defenses in the liver of HFD-fed rats. Furthermore, HMG-CoA reductase and lipase were suppressed by MEACL. In conclusion, this study shows the potential effect of MEACL to ameliorate hyperlipidemia and oxidative stress in HFD-fed rats. It prevented hepatic lipid accumulation and exerted an inhibitory effect on HMG-CoA reductase and lipase.

## 1. Introduction

Cardiovascular diseases (CVDs) are responsible for the highest burden of disease globally [1]. They are the leading causes of death, morbidity, and health expenses in developed and developing countries, accounting for around 30% of the annual global mortality and 10% of worldwide health burden [2]. Hyperlipidemia is the increase in plasma lipids, including total cholesterol (TC) and triglycerides (TG), and represents one of the main factors leading to CVDs. It has also been reported to be the most widespread marker for susceptibility to atherosclerotic heart disease [3]. High levels of plasma lipids, mainly cholesterol, are a common feature of atherosclerosis, a condition in which arterial damage can lead to ischemic heart disease, myocardial infarction, and cerebrovascular coincidences [4]. Despite having several therapeutic measures, focus has now been given to establishing effective preventive strategies for detecting and controlling cardiovascular risk factors [5]. It has been estimated that, by 2030, more than 24 million people per year will suffer from cardiovascular problems [6]. Globally, approximately 12 million people die due to CVDs each year. Factors such as diet high in saturated fats and cholesterol, age, family history, hypertension, and lifestyle are of great significance, but the high level of cholesterol, particularly low-density lipoprotein cholesterol (LDL-C), is mainly responsible for the occurrence of CVDs [7].

Statins, fibrates, and bile acid sequestrants are the currently used classes of lipid-lowering agents. These synthetic agents possess beneficial effects along with their lipid-lowering action; however, their use might be associated with undesired side effects, including rhabdomyolysis, myopathy, and increased risk of gallstones [8]. Therefore, developing new lipid-lowering agents with high therapeutic value and no or minimum side effects is needed [9].

*Averrhoa carambola* (*A. carambola*) is found in America, Brazil, Australia, and Southeast Asia (Malaysia, southern China, Taiwan, and India). It has been widely used in traditional medicine to treat several aliments. *A. carambola* is widely cultivated on a commercial scale and represents one of the most important species in Malaysia [10]. Several phytochemical and pharmacological reports have suggested that the extracts of different parts of *A. carambola* are rich in flavonoids, saponins, alkaloids, tannins, proanthocyanins, vitamin C, and gallic acid, which possess antioxidant and specific healing properties [11,12]. Accordingly, consumption of *A. carambola* has been demonstrated to boost immunity and help remove toxins from the body. In addition, *A. carambola* extracts exhibit anti-inflammatory and antiulcerogenic properties [13] and ameliorate nonalcoholic hepatic steatosis [14] and diabetic nephropathy [15]. Our previous study indicated that methanolic extract of *A. carambola* leaf (MEACL) demonstrated antihyperlipidemic and antioxidant effects in poloxamer-407-induced hyperlipidemic rat model that were comparable to that of atorvastatin [16]. This has created an interest to evaluate the beneficial effect of *A. carambola*-standardized leaf extract in a model of high-fat diet (HFD)-fed rats, pointing to its antihyperlipidemic and antioxidant efficacies.

## 2. Materials and Methods

### 2.1. Chemicals, Reagents, and Standards

Isopropanol, sodium citrate, disodium hydrogen phosphate, formalin, hydrogen peroxide, potassium chloride, sodium hydroxide, tris (hydroxyl methyl) aminomethane-HCl, and carboxymethylcellulose (CMC) were purchased from Sigma-Aldrich (St Louis, USA). Atorvastatin and normal saline were supplied by Ranbaxy (Sdn. Bhd., Malaysia). Cholic acid and sodium pentobarbitone were procured from Sigma-Aldrich. Analytical-grade methanol and chloroform were purchased from R & M Chemicals (Essex, UK). Acetic acid was purchased from Merck (Darmstadt, Germany). Cholesterol and commercial food were bought from Animal Research and Service Centre (ARASC, USM, Malaysia), while margarine was obtained from Tesco (Penang, Malaysia).

### 2.2. Collection of A. Carambola and Extract Preparation

The freshly harvested leaves of *A. carambola* were collected from the main campus of University Sains Malaysia (USM, Penang, Malaysia). A voucher specimen, catalogue No. 11238, was deposited in the herbarium of School of Biological Sciences (USM, Malaysia). The leaves were thoroughly cleaned, oven-dried at 45 °C, and pulverized into fine powder using a grinder. The powdered plant material was extracted in methanol (powder: solvent ratio of 1:6 w/v) by maceration for 9 consecutive days. Fresh solvent was replenished every 72 h. The methanolic extract was filtered through a cotton plug followed by Whatman No. 1 filter paper and concentrated in vacuo at 45 °C until dryness. The dried extract was freeze-dried and kept in the fridge prior to use [16,17].

### 2.3. Standardization and Quantification of Apigenin in A. Carambola Extract Using High Performance Liquid Chromatography (HPLC)

#### 2.3.1. Development and Validation of the HPLC Method

HPLC was used to standardize and quantify apigenin in MEACL. The working solution of extract was prepared in methanol at 10 mg/mL concentration. The working sample solutions were filtered using 0.45 µm polytetrafluoroethylene (PTFE) syringe filter and kept at −20 °C for HPLC analysis. Apigenin was used as a marker compound, and the stock solution was prepared in methanol at concentration of 500 µg/mL. Serial dilutions were prepared from the working standard solution using the mobile phase to a concentration of 50, 20, 5.0, 2.5, 1.25, and 0.625 µg/mL. Apigenin was used to assess the linearity, recovery, accuracy, and precision. All the prepared standard solutions were kept at −20 °C to be used for HPLC analysis.

The Agilent 1200 Series HPLC system is composed of an auto sampler–injector, a quaternary pump, and diode array detector (DAD). Data collection and integration were carried out using Agilent ChemStation software. The chromatographic separation was done on Hypersil GOLD C4 column (250 × 4.6 mm, 5 µm, Thermo Scientific, Waltham, MA, USA). The sample injection volume was 20 µL, which was eluted by a gradient mobile phase consisting of 0.1% aqueous formic acid (A) and acetonitrile (B), as illustrated in Table 1. The temperature was set at 40 °C with a flow rate of 1 mL/min, and the UV detection was at 340 nm (Figure S1).

The linearity of the method was evaluated by plotting the concentration against the peak area to establish the calibration curve of the marker compound. The calibration curve of apigenin was structured by plotting the concentration versus peak area in a range of 50–0.625 µg/mL (Figure S2). The linearity was assessed by linear regression analysis (correlation coefficient (*R^2^*) and standard deviation (SD). The limit of detection (LOD) is known as the lowest concentration of analyte that can be detected in the HPLC system. It is normally determined from the analyte peak, which gives the signal-to-noise ratio of 3:1. The limit of quantification (LOQ) is defined as the lowest concentration with acceptable accuracy and precision, which can be used for quantification purposes and measured at the signal-to-noise ratio of 8:1 [18].

The accuracy test is used to determine the closeness between the actual value and the value obtained from the HPLC analysis, while precision test is used to determine the repeatability and reproducibility of the HPLC method to produce an acceptable range of values with a relative standard deviation (RSD) in the range of 5% or below [19]. In the current study, validation of the HPLC method was carried out for five days to evaluate the within-day and between-day accuracy and precision using the standard solutions. The within-day accuracy and precision were evaluated by injecting the standard working solutions into the HPLC five times on the same day. The between-day accuracy and precision analysis were carried out by injecting the standard working solutions one time/day for five consecutive days (*n* = 5) [20].

The aim of a recovery study is to determine whether the extraction process causes any loss of the authentic matrix by comparing the amount of the authentic compound in raw samples and the spiked samples [21]. In this study, the recovery was carried out by spiking 5.0 g dried powder of *A. carambola* leaf with three different concentrations of apigenin standard solutions. The concentrations of apigenin standard solutions were 50.00, 5.00, and 0.625 µg/mL. The spiked powder of *A. carambola* leaf was then extracted with 100 mL of 95% methanol for six days. The solvent was replaced three times every two days. The same quantity of the powder without spike was also extracted as a control. The extracts were reconstituted in methanol and filtered using a 0.45 µm syringe filter (Pall Co. Ann Arbor, USA), and 20 µL of these samples was injected for HPLC analysis. The calculation of extraction recovery value of compound was done by dividing the percentage of concentration of the spiked compound obtained after the extraction over that of the actual amount of the spiked compound. The extraction recovery of the marker was calculated as a percentage using the following equation as previously described [22]:

(1) $$\%\;\mathrm{Recovery}\;=\;\frac{\left(\mathrm{value}\;\mathrm{obtained}\;\mathrm{of}\;\mathrm{sample}\;-\;\mathrm{value}\;\mathrm{obtained}\;\mathrm{of}\;\mathrm{control}\right)}{\left(\mathrm{spiked}\;\mathrm{value}\right)}\;\times \;100$$

#### 2.3.2. Analysis and Standardization of Apigenin in MEACL

Analysis of MEACL using HPLC was carried out using the developed and validated method in order to determine the contents of apigenin in MEACL. Apigenin was used as a marker compound for standardization, and a calibration curve was used for quantification of apigenin in MEACL.

### 2.4. Animals and Treatments

Male Sprague Dawley (SD) rats, weighing 180–250 g and obtained from the Animal Research and Service Centre (ARASC, USM, Malaysia), were used in this study. The animals were kept at the animal transit room of the School of Pharmaceutical Sciences, USM, and maintained at 24 ± 2 °C, 47 ± 12% relative humidity, and 12 h light/dark cycle with free access to food and tap water. The animals were allowed to acclimatize in the new environment for a period of 7 days prior to experimentation. Experimental procedures were in accordance to institutional guidelines, and approval was obtained from the Animal Ethics Committee at USM (Penang, Malaysia) [Approval number: USM/Animal Ethics Approval/2012/(77)(387)].

#### 2.4.1. Induction of Hyperlipidemia

The HFD was prepared by mixing powdered commercial standard food (12% protein, 35.68% fat, 35.64% carbohydrates) [23] with margarine (energy: 3700 kg/100g, milk fat: 99.85 g, and moisture: 0.15 g), cholesterol, and cholic acid in a ratio of 88.5:10:1:0.5 (*w*/*w*/*w*/*w*). The ingredients were mixed well and made into small pellets. The pellets were then dried in the oven at 80 °C for 2 h, cooled at room temperature, and kept at 4 °C prior to use. Hyperlipidemia was induced by feeding the HFD for 10 days before starting the experiments. Hyperlipidemia was confirmed by measuring TC and TG using a commercially available lipid analyzer meter (Cardio Chek, Germany), and hyperlipidemic rats were selected for further experiments.

#### 2.4.2. Experimental Design

Thirty-six SD rats were divided into 6 groups (6 animals/group) as follows:

Group I: normal rats received normal commercial diet and were given 1% CMC (5 mL/kg) orally for 5 weeks.

Group II: normal rats received 1000 mg/kg MEACL dissolved in 1% CMC orally for 5 weeks.

Group III: HFD-fed rats received HFD and 1% CMC orally for 5 weeks.

Group IV: HFD-fed rats received HFD and atorvastatin (20 mg/kg) orally for 5 weeks.

Group V: HFD-fed rats received HFD and 250 mg/kg MEACL orally for 5 weeks.

Group VI: HFD-fed rats received HFD and 500 mg/kg MEACL orally for 5 weeks.

Group VII: HFD-fed rats received HFD and 1000 mg/kg MEACL orally for 5 weeks.

Blood samples were withdrawn from the tail vein before treatment and after 7, 14, 21, and 28 days for determination of TC and TG. Daily food intake and body weight changes were recorded throughout the experiment. At the end of the experimental period, overnight-fasted rats were anesthetized with sodium pentobarbitone (60 mg/kg), and blood was collected for serum separation.

### 2.5. Determination of Serum Lipids and Atherogenic Index (AI)

Serum TC was measured according to the manufacturer’s protocol (Thermo Fisher Scientific, Waltham, MA, USA), whereas TG was determined using colorimetric assay kit (Cayman Chemical, Ann Arbor, MI, USA). High-density lipoprotein cholesterol (HDL-C) and LDL-C levels were measured by enzymatic colorimetric method using ARCHITECT C4000 Biochemistry Analyzer at the pathology laboratory in Lam Wah Ee Hospital (Penang, Malaysia). Levels of very low-density lipoprotein cholesterol (vLDL-C) and AI were calculated using the following formulas [24]:

vLDL-C = TG/2.22(2)

AI = (TC − HDL)/HDL(3)

### 2.6. Determination of the BMI

BMI is defined as the ratio between body weight in g and length in cm^2^ in case of rats. The rat’s length was measured from the nose to the tail base [25]. The BMI was calculated according to the following formula:

BMI = weight(g)/length(cm)^2^ (4)

### 2.7. Histopathology

Liver samples were collected from all animals, fixed for 48 h in 10% neutral-buffered formalin. The fixed samples were prepared by dehydration through increasing concentrations of alcohol, followed by clearing using xylene and embedding in paraffin. Sections of 5 μm thickness were prepared using a rotary microtome and stained with hematoxylin and eosin (H&E), mounted onto glass slides, and cover-slipped for microscopic observations.

### 2.8. Determination of HMG-CoA Reductase and Pancreatic Lipase Activities

The activity of HMG-CoA reductase (EC 1.1.1.34) was determined using a commercially available kit purchased form Sigma-Aldrich (St Louis, MO, USA). The assay is based on the oxidation of nicotinamide adenine dinucleotide phosphate (NADPH) by the catalytic subunit of HMG reductase in the presence of the substrate HMG-CoA. Pravastatin was used as reference standard. The assay was done at 37 °C according to the manufacturer’s protocol [26].

In addition, the effect of MEACL on pancreatic lipase activity (EC 3.1.1.3) was measured using 4-methyl umbelliferone oleate (4-MUO) as described previously with some modification [27]. The amount of 4-MUO released by lipase was measured using a microplate reader (TECAN Infinite Pro^®^ M200, Mannedorf, Switzerland) at an excitation wavelength of 320 nm and an emission wavelength of 450 nm. Results are expressed as percentage of inhibition.

### 2.9. Determination of Lipid Peroxidation and Antioxidants

At the end of the study, liver tissues were collected for preparation of 10% liver homogenate (*w*/*v*). The homogenates were prepared in ice-cold 0.1 M Tris-HCl buffer (pH 7.4) and homogenized using a homogenizer at 1000 rpm for 3 min. Subsequently, the homogenates were centrifuged at 5000× rpm, and the supernatant was collected and kept at −80 °C for analysis. Protein concentration was determined as previously described [28]. Thiobarbituric acid reactive substances (TBARS) [29], reduced glutathione (GSH) [30], superoxide dismutase (SOD) [31], glutathione peroxidase (GPX) [32], and catalase (CAT) [33] were determined in the liver homogenate.

### 2.10. Determination of Hepatic and Fecal Lipids

Total lipids were extracted from the liver samples using chloroform: methanol mixture (2:1, *v*/*v*) [34]. A 100 mg tissue was homogenized in 500 μL of phosphate buffer (pH 7.4), centrifuged at 9000× rpm for 10 min at 4 °C, and the supernatant was transferred to a new tube. A volume of 1 mL chloroform/methanol (2:1 *v*/*v*) was added to the supernatant, and the mixture was sonicated for 30 min and allowed to stand for 1 h. The chloroform phase was collected and allowed to stand in the fume hood until dryness. Subsequently, 1000 μL isopropanol was added for reconstitution, and samples were vortexed prior to analysis. Fecal samples were collected before treatment during the last 48 h of the experiment, and lipids were extracted using chloroform/methanol solution. Levels of TC and TG were measured in feces samples using commercial assay kits supplied by Thermo Fisher Scientific (Waltham, MA, USA).

### 2.11. Determination of Fecal Bile Acids

The extraction of bile acids from feces was performed as previously described [35]. In brief, fecal samples were collected for 48 h prior to the end of the experiment, dried, weighed, and frozen at −70 °C. Hundred milligrams was extracted overnight with 1 mL chloroform/methanol (2:1, *v*/*v*) and 2 mL KCl (3.7 g/L) was then added to remove the contaminants. The sample was then centrifuged at 1500× *g* for 10 min. The upper layer was removed, evaporated, and dissolved in 1 mL methanol (50%). A 20 μL sample, 250 μL NAD (2 mmol/L) dissolved in phosphate buffer (pH 10.5), and 30 μL of 3α-hydroxysteroid dehydrogenase (0.1 IU per sample) were mixed and incubated for 30 min at room temperature. The absorbance was measured at 340 nm, and the results were expressed as μg bile acid per 100 mg of dry feces [36].

### 2.12. Statistical Analysis

The data were presented as mean ± standard error of the mean (SEM) and analyzed using one-way ANOVA followed by Tukey’s post hoc test on GraphPad Prism 7 (GraphPad Software, La Jolla, CA, USA). A *p* value < 0.05 was considered significant.

## 3. Results

### 3.1. Standardization and Quantification of Apigenin in MEACL

#### 3.1.1. HPLC Validation Method

The chromatographic separation was carried out using Hypersil GOLD C4 column (250 × 4.6 mm, 5 µm, Thermo Scientific, USA) at 1 mL/min. The calibration curve for the standard was linear in the range of 0.625–50 µg/mL. The LOD and LOQ for apigenin were 0.15 and 0.63 µg/mL, respectively (Table S1). The recovery values of apigenin in MEAC are presented in Table 2. The recovery values of the studied concentrations ranged between 99.96% and 104.00%, while the RSD and CV values ranged between 3.10% and 3.99% and 2.94 and 4.15, respectively.

Results of within-day and between-day precision and accuracy for apigenin measurements in MEACL are illustrated in Table 3. The results of accuracies and precisions of within-day ranged between 95.08% and 104.07% and 1.01% and 4.03%, respectively, while the accuracies and precision of between-day ranged between 95.24% and 104.92% and 1.32% and 5.04%, respectively.

#### 3.1.2. HPLC–UV Analysis of MEACL

**Figure 1:**
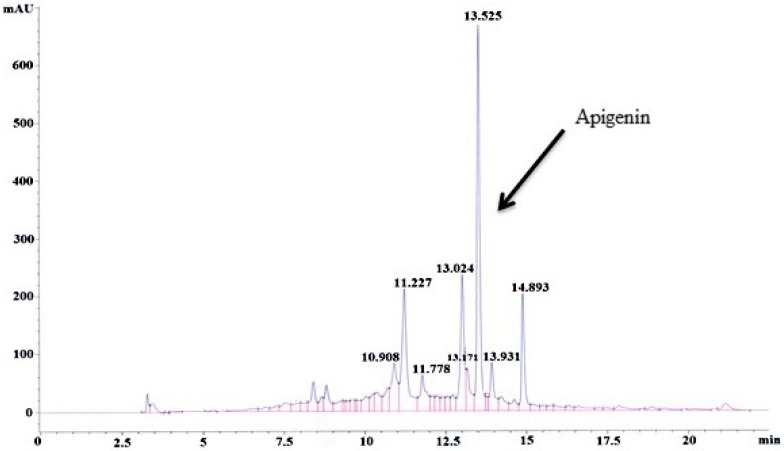
HPLC chromatogram for methanolic extract of *Averrhoa carambola* leaf (MEACL) at 5 mg/mL.

The developed and validated HPLC method was used to determine the content of apigenin in MEACL (Figure S3). The calculated content of apigenin in MEACL was found to be 4.83 µg/mg (Table S2). The HPLC chromatogram for MEAC at 5 mg/mL is illustrated in Figure 1.

### 3.2. Effects of MEACL on Body Weight, Body Mass Index, Food Intake, and Relative Organ Weight

**Figure 2:**
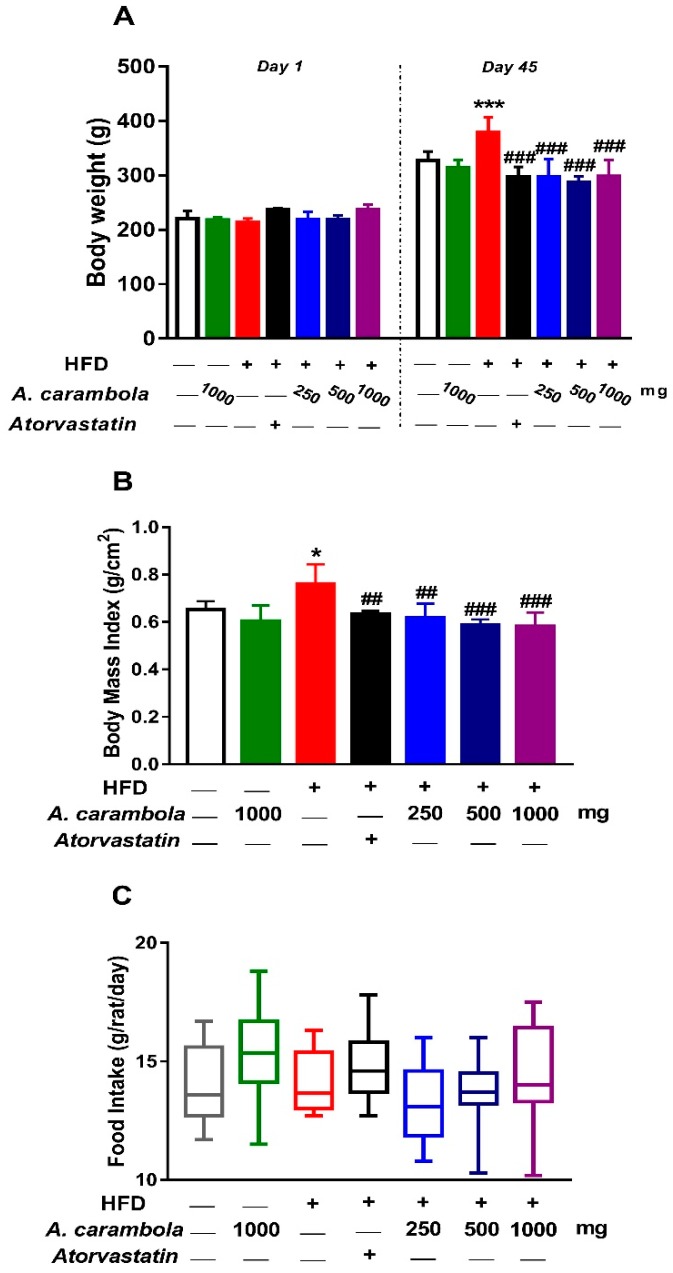
Body weight (**A**), body mass index (**B**), and food intake (**C**) of high-fat diet (HFD)-induced hyperlipidemic rats after treatment with various doses of MEACL for five weeks. Values are represented as mean ± SEM, *n* = 6. * *p* < 0.05, *** *p* < 0.001 compared to normal control. ## *p* < 0.01, ### *p* < 0.001 compared to hyperlipidemic control.

The body weight of the rats at the beginning of the experiment ranged between 212.37 and 236.58 g, while body weight on day 45 following treatment ranged between 286.03 and 360.98 g. The comparison between the weights of the rats after 45 days of treatment and their weights on day 1 in the corresponding groups was analyzed using paired *t*-test. The results showed statistically significant increase in all experimental groups (*p* < 0.001) (Figure 2A).

The BMI of the rats in all groups was estimated at the end of the experiment. The length of rats ranged between 20.2 and 24.2 cm, while their weight ranged between 258.8 and 397.4 g. A significant increase in the BMI was observed between the hyperlipidemic control and the normal control groups. In contrast, statistically significant decrease in BMI was detected at 250 and 500 mg/kg (*p* < 0.01) and 1000 mg/kg (*p* < 0.001) extract-treated animals compared to the hyperlipidemic control group (Figure 2B).

The average daily food intake was estimated as g/rat/day for each group. Figure 2C shows the average daily food intake among treated and untreated groups, which ranged between 13.3 and 14.8 g/day/rat. Average daily food intake was greater in the atorvastatin group, while it was lesser in the hyperlipidemic group treated with 250 mg/kg MEACL. However, there were no statistical differences in the average daily food intake between the hyperlipidemic control group and normal control or MEACL-treated groups (Figure 2C).

Findings on the relative liver weight of the animals were similar to that of the BMI. The hyperlipidemic control group presented a significant increase (*p* < 0.01) in the relative liver weight compared with the normal control group, while a significant decrease was observed in the treated groups (500 and 1000 mg/kg MEACL) (*p* < 0.05) compared to the hyperlipidemic control group. Adipose tissue weight of high dose of MEACL showed a statistical decrease in comparison with the hyperlipidemic control group (*p* < 0.05). There were no statistical differences in the relative organ weight for the other isolated organs among the different experimental groups (Table 4).

### 3.3. Antihyperlipidemic Effect of MEACL in HFD-Fed Rats

At the beginning of the study, TC and TG levels of all groups were within the normal range without any significant difference. After 10 days induction using HFD, all groups given HFD showed statistically significant increase in lipid parameters compared to the normal control group (*p* < 0.001). Hyperlipidemic control group showed statistically significant increase in TC and TG levels when compared with normal control group throughout the study period of five weeks.

**Figure 3:**
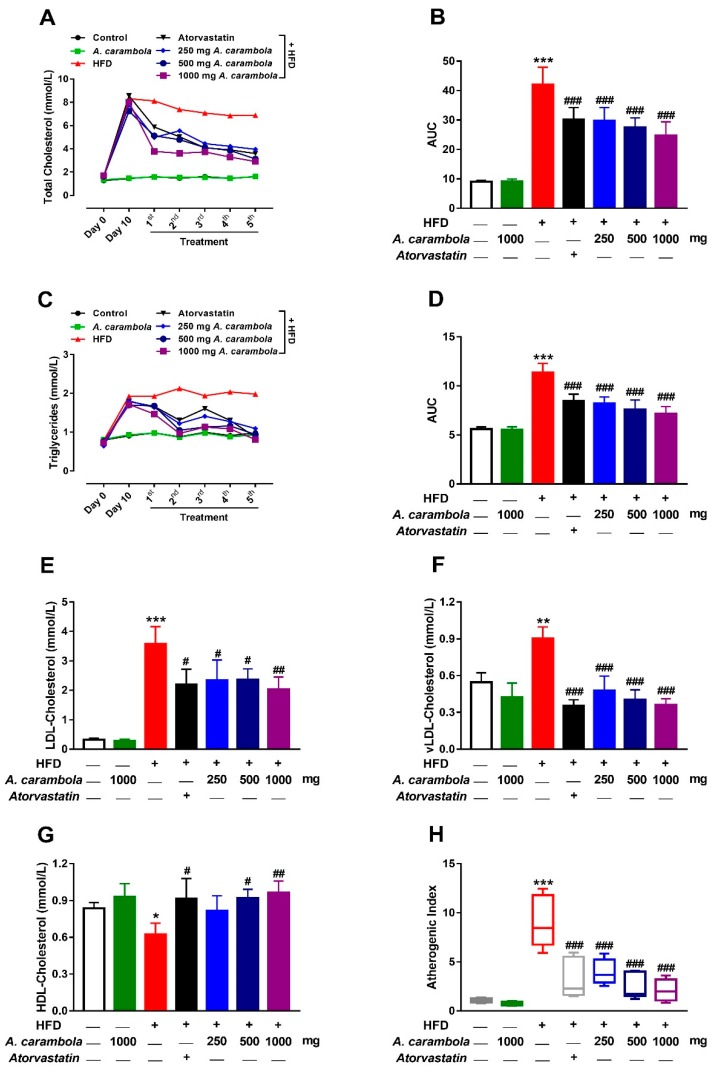
Effect of MEACL on levels of total cholesterol (TC) (**A,B**), triglycerides (TG) (**C,D**), low-density lipoprotein cholesterol (LDL-C) (**E**), very low-density lipoprotein cholesterol (vLDL-C) (**F**), high-density lipoprotein cholesterol (HDL-C) (**G**), and atherogenic index (AI) (**H**) of HFD-induced hyperlipidemic rats after five weeks of treatment. AUC, area under the curve. Values are expressed as mean ± SEM, *n* = 6. * *p* < 0.05, ** *p* < 0.01, *** *p* < 0.001 compared to normal control. # *p* < 0.05, ## *p* < 0.01, ### *p* < 0.001 compared to hyperlipidemic control.

A statistically significant reduction was observed in the serum levels of TC after five weeks of treatment with MEACL at all the three tested doses (*p* < 0.001) when compared with the hyperlipidemic control group. A significant decrease in TC levels was also noted after four weeks of treatment with 250 (*p* < 0.05), 500 (*p* < 0.01), and 1000 (*p* < 0.001) mg/kg. Similarly, atorvastatin-treated animals had a significant decrease in TC levels after four and five weeks of treatment (*p* < 0.01 and *p* < 0.01, respectively). Only the high dose of MEACL showed statistically significant lowering effect on TC levels after the first, second, and third weeks of treatment when compared to the hyperlipidemic control group, whereas atorvastatin did not exhibit any statistically significant lowering effect during the same time period (Figure 3A,B).

On the other hand, a significant reduction was observed in the serum level of TG after five weeks of treatment with MEACL at all three tested doses (*p* < 0.001) when compared with the hyperlipidemic control group. Meanwhile, the atorvastatin group showed a significant decrease in TG levels after four and five weeks of treatment (*p* < 0.05 and *p* < 0.001, respectively) in comparison with the hyperlipidemic control group (Figure 3C,D).

A statistically significant increase was detected between normal control versus hyperlipidemic control group in LDL-C levels, vLDL-C, and AI (Figure 3E–G, respectively), but a significant decrease was observed in HDL-C levels (Figure 3H). The group treated with the high dose (1000 mg/kg) exhibited significant decrease in LDL-C levels and significant increase in HDL-C levels compared to the hyperlipidemic control group (*p* < 0.01). Likewise, the atorvastatin and 500 mg/kg groups exhibited significant changes (*p* < 0.05; Figure 3E,G). Meanwhile, significant reduction was detected in vLDL-C and AI levels for the hyperlipidemic control group with atorvastatin and the three groups with the tested doses of MEACL (*p* < 0.001; Figure 3F,H).

### 3.4. MEACL Prevents HFD-Induced Histopathological Alterations

**Figure 4:**
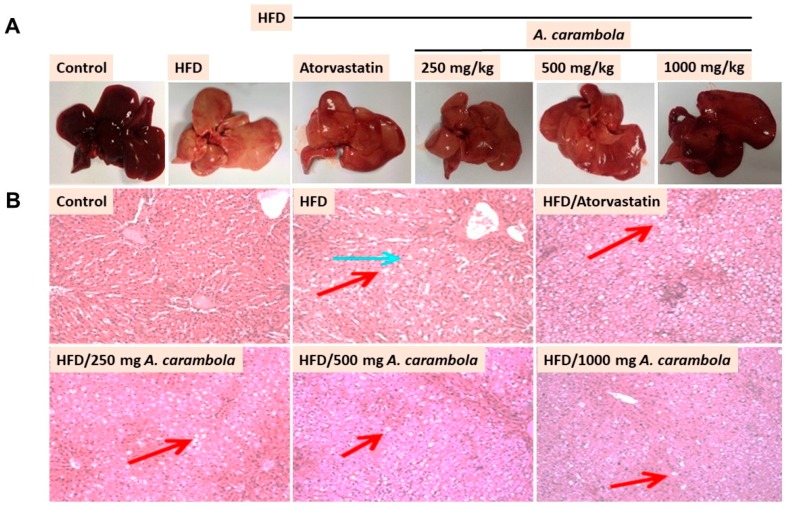
Effect of MEACL on rat liver gross and histology of HFD-induced hyperlipidemic rats as assessed by hematoxylin and eosin (H&E) staining. (**A**) Gross appearance of liver, (**B**) microscopic appearance of liver for normal control, hyperlipidemic control (severe macrovesicular steatosis, severe coagulation necrosis of hepatocyte, and severe cholestasis), and HFD-fed rats treated with atorvastatin (macrovesicular steatosis, lobular inflammation with multiple small foci, and mild cholestasis), 250 mg/kg MEACL (macrovesicular steatosis, lobular inflammation with multiple small foci, and mild cholestasis), 500 mg/kg MEACL (macrovesicular steatosis, lobular inflammation with multiple small foci, and mild cholestasis), and 1000 mg/kg MEACL (macrovesicular steatosis, lobular inflammation with few small foci, and no fibrosis or cholestasis). 100X magnification. Red arrow indicates macrovascular steatosis, and blue arrow indicates the coagulation necrosis of hepatocytes.

Histopathological examination of liver samples revealed macrovesicular steatosis, lobular inflammation, fibrosis, and cholestasis in HFD-fed rats. Figure 4A shows the gross appearance of liver samples, while Figure 4B shows the microscopic appearance of liver tissues. The normal control group showed normal hepatic architecture with distinct hepatic cells, sinusoidal spaces, and central veins. Hepatocytes with polygonal cells, well-preserved cytoplasm, and nucleus with prominent nuclei were present. No macrovesicular steatosis, lobular inflammation, fibrosis, or cholestasis was seen in the normal control group (Figure 4).

In contrast, the histological examination of the hyperlipidemic control group showed loss of architecture, severe macrovesicular steatosis in the periportal and midzone areas, severe coagulation necrosis of lobular hepatocytes, and severe cholestasis. The atorvastatin-treated group and groups treated with 250 and 500 mg/kg MEACL exhibited severe macrovesicular steatosis in the periportal and midzone areas, chronic lobular inflammation and mild cholestasis. Histopathological examination of the animals treated with 1000 mg/kg MEACL showed some evidence of recovery with no fibrosis or cholestasis when compared with the hyperlipidemic control group, which showed severe macrovesicular steatosis in the periportal and midzone areas and few foci of lobular inflammation (Figure 4B).

### 3.5. Inhibitory Activity of MEACL on HMG-CoA Reductase and Pancreatic Lipase

**Figure 5:**
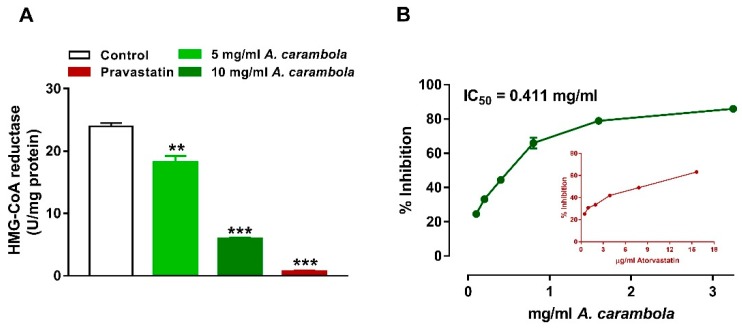
Inhibitory activity of MEACL on HMG-CoA reductase (**A**) and pancreatic lipase (**B**). ** *p* < 0.01, *** *p* < 0.001 compared to Control.

A statistically significant dose-dependent inhibitory effect of MEACL on HMG-CoA reductase was observed at both 5 and 10 mg/mL concentrations (18.1 and 5.91 units/mg, respectively) compared with the control (*p* < 0.05 and *p* < 0.01, respectively). Pravastatin showed a larger reduction in the HMG-CoA reductase enzyme activity (*p* < 0.001) (Figure 5A). The inhibitory activity of MEACL was measured against pancreatic lipase. The IC_50_ value of atorvastatin was found to be 6.8 ± 0.31 μg/mL, while the IC_50_ of the MEACL was 0.411 ± 0.023 mg/mL (Figure 5B). The inhibitory activity of MEACL was found to be stronger when compared to that of the atorvastatin.

### 3.6. Effect of MEACL on Levels of Liver TC, TG, Fecal Cholesterol, and Fecal Bile Acids of HFD-induced Hyperlipidemic Rats

**Figure 6:**
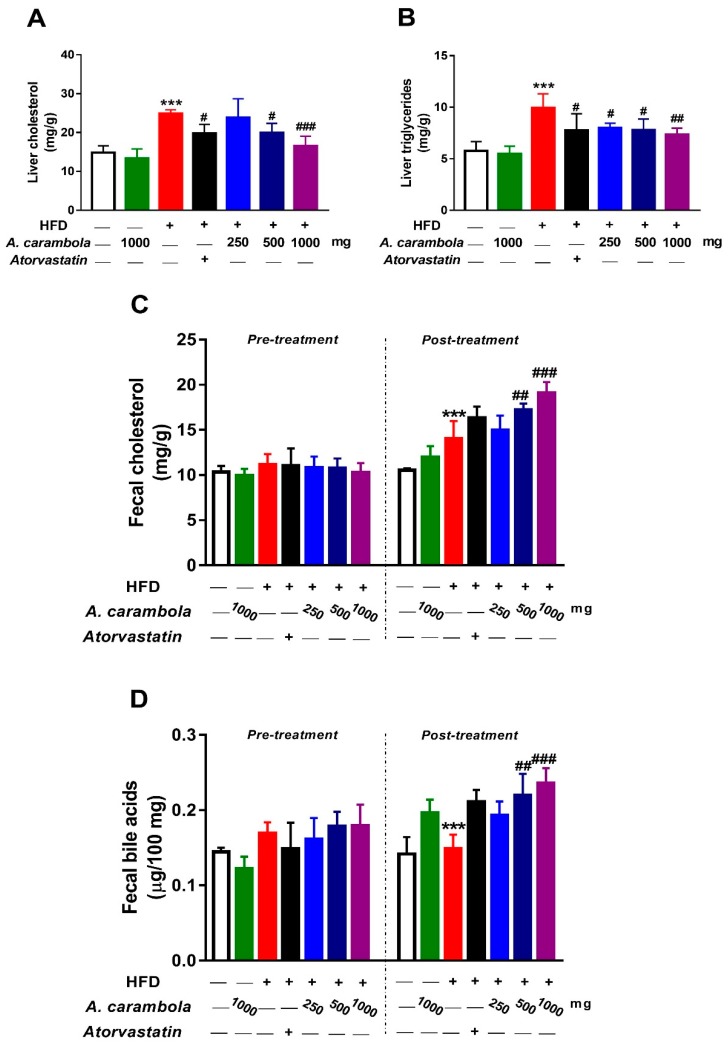
Effect of MEACL on levels of liver total cholesterol (**A**), triglycerides (**B**), fecal cholesterol (**C**), and fecal bile acids (**D**) in HFD-induced hyperlipidemic rats. Values are represented as mean ± SEM, *n* = 6. *** *p* < 0.001 compared to normal control. # *p* < 0.05, ## *p* < 0.01, ### *p* < 0.001 compared to hyperlipidemic control.

Figure 6 shows that the liver TC and TG levels in the hyperlipidemic control group showed significant increase when compared with the normal control (*p* < 0.001). A 1000 mg/kg dose of MEASL significantly lowered the hepatic TC and TG levels in comparison with the hyperlipidemic control group (*p* < 0.001 and *p* < 0.01, respectively). The atorvastatin-treated and the 500 mg/kg MEACL-treated hyperlipidemic groups had significantly lower liver TC and TG levels (*p* < 0.001). A significant decrease in TC levels was observed at 250 mg/kg, but no significant change was noted in liver TG levels (Figure 6A,B).

Fecal TC and bile acid levels of HFD-induced chronic hyperlipidemic rats were compared with the hyperlipidemic control group after five weeks of treatment with MEACL using the ANOVA test. The hyperlipidemic control group showed statistically significant increase in fecal TC levels when compared with the normal control group (*p* < 0.001). Hyperlipidemic animals treated with 500 and 1000 mg/kg MEACL exhibited significant increases in fecal TC and bile acid levels compared with the hyperlipidemic control group (*p* < 0.01 and *p* < 0.001, respectively), whereas, groups treated with atorvastatin and 250 mg/kg did not show any statistically significant increase in fecal TC or bile acid levels when compared with the hyperlipidemic control group (Figure 6C,D).

### 3.7. Antioxidant Activity of MEACL in HFD-Induced Rats

Levels of GSH, GPx, SOD, and catalase in liver homogenates of the hyperlipidemic control group exhibited statistically significant decrease in comparison with their levels in the normal control group, while malondialdehyde (MDA) levels in the hyperlipidemic control group showed statistically significant increase when compared with levels in the normal control group.

**Figure 7:**
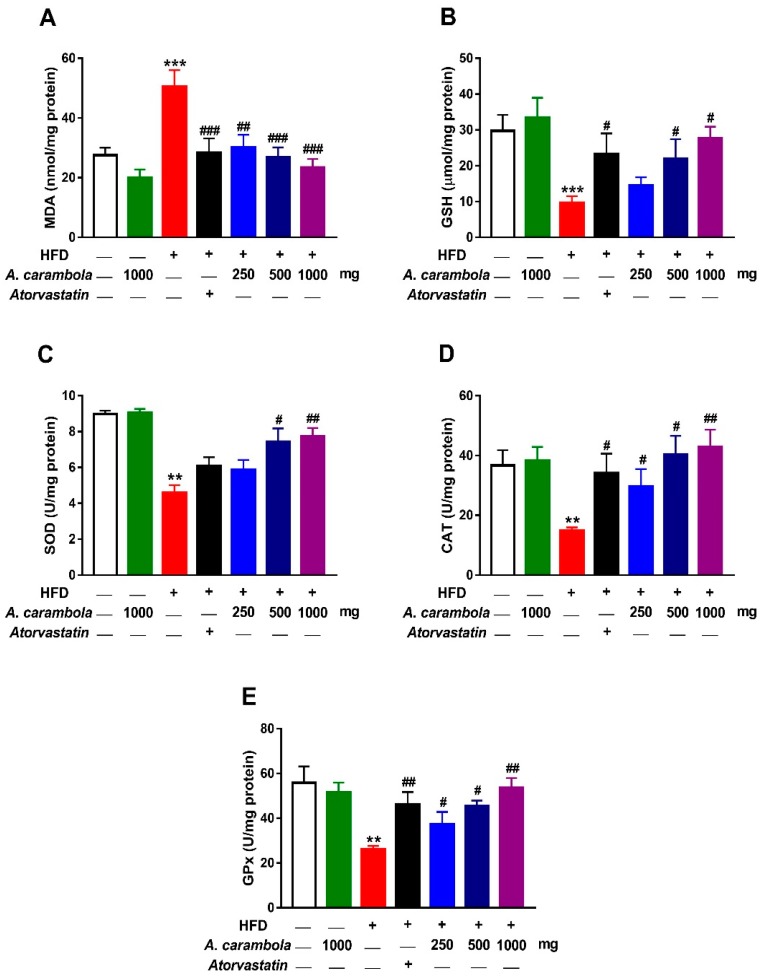
Effect of MEACL on selected in vivo antioxidant parameters in the liver of HFD-induced chronic hyperlipidemic rats. MEACL reduced hepatic MDA (**A**), and increased GSH (**B**), SOD (**C**), CAT (**D**) and GPx (**E**) in HFD-fed rats. MDA, malondialdehyde; SOD, superoxide dismutase; GSH, reduced glutathione; GPx, glutathione peroxidase. Values are expressed as mean ± SEM, *n* = 3. ** *p* < 0.01, *** *p* < 0.001 compared to normal control. # *p* < 0.05, ## *p* < 0.01, ### *p* < 0.001 compared to hyperlipidemic control.

A significant decrease in MDA levels (Figure 7A) were detected in all treated groups compared to the hyperlipidemic control group. The higher dose (1000 mg/kg) of MEACL showed significant increase in levels of GSH (*p* < 0.05; Figure 7B), SOD (*p* < 0.001; Figure 7C), catalase (*p* < 0.01; Figure 7D), and GPx (*p* < 0.01; Figure 7E) when compared with the hyperlipidemic control group. Similarly, the 500 mg/kg dose exhibited statistically significant increase in levels of GSH, GPx, SOD, and catalase (*p* < 0.05) when compared with the hyperlipidaemic control group.

It was noted that a 20 mg/kg dose of atorvastatin showed significant increase in levels of GSH, CAT (*p* < 0.05), and GPx (*p* < 0.01), whereas no statistically significant increase was noted in SOD levels (Figure 7C).

## 4. Discussion

Cardiovascular risk evaluation has been found to be valuable in minimizing and controlling various CVDs [1]. Hyperlipidemia is a major risk factor for coronary heart disease. Thus, it becomes one of the most significant public health problems [37]. The use of plants in the treatment of dyslipidemia is one of the ongoing approaches worldwide as they have broad pharmacological effects through various mechanisms.

The process of extraction of bioactive compounds from plant materials is the initial step in the exploitation of phytochemicals toward the preparation of dietary supplements, pharmaceuticals, cosmetic products, and food ingredients [38]. Methanol is a well-known universal solvent, possessing the capability to extract semipolar bioactive compounds [16]. In this context, a prior study revealed that MEACL has the most potent antihyperlipidemic and antioxidant activities, which are associated with phenolic and flavonoid contents, in particular high flavonoid contents [16]. Accordingly, MEACL was chosen as the candidate for further investigations on chronic hyperlipidemia model using HFD-fed rats.

The literature has reported that apigenin is a flavone molecule that has potential biological activities, such as antioxidant activity, tissue protective effect, and inhibitory activity on hepatic cholesterol biosynthesis [39,40]. In addition, apigenin has been revealed to help in improving cardiovascular conditions, stimulating immune system, reducing plasma LDL-C levels, inhibiting platelet aggregation, and reducing cell proliferation [41]. Consequently, apigenin was used as a marker compound for standardization and quantification of MEACL. The recovery data for the apigenin compound was satisfactory, meaning that the extraction process did not result in any substantial loss of this compound. The results of accuracy of within-day and between-day indicate that the method is reliable, repeatable, and reproducible. The outcome of LOD of apigenin in this study was 0.15 µg/mL, which is higher than 1.94 ng/mL [42] but lower than 2.5 µg/mL [43], while the results of LOQ was 0.625 µg/mL, which is higher than 31.45 and 10 ng [42,44].

In the current study, HFD significantly increased the TC and TG levels when given to the experimental animals for 10 days during the pretreatment period. Oral administration of different doses of MEACL produced significant reductions in lipids and significant increase in HDL-C levels to near normal. The 1000 mg/kg dose of MEACL was the most effective at reducing serum levels of TC and TG after five weeks of treatment when compared with the other two doses. The antihyperlipidemic activity of 500 and 1000 mg/kg MEACL was comparable to that of atorvastatin. HDL-C plays an important role in transferring cholesterol and cholesterol esters from tissues and cells to the liver, where they are metabolized to bile acids. Thus, HDL-C achieves an essential function of decreasing cholesterol levels in blood and peripheral tissues and prevent atherosclerosis plaque formation in the aorta [45]. Meanwhile, TG plays a key role in maintaining normal lipid metabolism through regulation of lipoprotein interactions. Increased serum TG levels were interrelated with an amplified rate of coronary artery disease [17]. On the other hand, the oxidation of LDL-C in the artery walls by oxygen free radicals leads to the production of oxidized LDL-C, which attracts the macrophage scavenger of the immune system. These macrophages accumulate at the arterial wall after ingestion of oxidized LDL-C particle, and their concentration is associated with atheromatous arteriosclerosis plaques. Higher TC, TG, and LDL-C levels and lower HDL-C levels are risk factors for atherosclerosis. These processes may lead to many complications, such as coronary heart disease, ischemic stroke, and occlusive arterial disease of the lower limbs [46].

Investigation of the possible mechanism behind the antihyperlipidemic activity of MEACL showed the involvement of HMG-CoA reductase and pancreatic lipase, two key enzymes regulating the production and metabolism of cholesterol and lipids [47]. Inhibition of HMG-CoA reductase enzyme is the prime step in treating hyperlipidemia. As a result of this inhibition, a decrease in the endogenous cholesterol synthesis and other products downstream of mevalonate can be achieved [47]. Pancreatic lipase is responsible for the hydrolysis of TG into monoglycerides and fatty acids [48]. The hydrolysis step is crucial because these particular lipids should first be hydrolyzed before undergoing absorption through the lining of the intestine [49]. The effectiveness of hydrolysis of TG relies on adequate availability of bile salts provided by the liver [49]. Pancreatic lipase is considered a key enzyme responsible for TG absorption in the small intestine, and the inhibition of this enzyme could be a key approach for controlling hyperlipidemia and obesity through suppression and delay of digestion and absorption of TG [47]. In the present study, dose-dependent inhibitory effects of MEACL on HMG-CoA reductase and pancreatic lipase were observed, which could be one of the possible mechanisms behind the antihyperlipidemic efficacy of this plant extract.

Significant changes were detected in body weight, relative liver weight, and BMI of the treated groups compared with the hyperlipidemic control group. Despite these changes, daily food consumption was similar in all groups. These results illustrate that MEACL had the capability to lower lipid levels in HFD-fed rats, suggesting an antihyperlipidemic efficacy. In the histopathological observation, oral treatment with MEACL showed dose-dependent effects on the liver of HFD-fed rats. Consequently, the antihyperlipidemic effect of MEACL was evident by attenuation of liver histological features produced by HFD in the experimental rats, as apparent in both gross and microscopic examination.

Accumulating evidence indicates that oxidative stress is an early event in the evolution of hyperlipidemia [50,51]. Hyperlipidemia is associated with oxidative modification of LDL-C, protein glycation, and glucose autoxidation [50]. In addition, increased cholesterol levels may lead to an elevation in cholesterol pool, which results in altered physical properties of cell membrane, facilitating the leakage of reactive oxygen species (ROS) from the mitochondrial electron system or the activation of NADPH oxidase, eventually culminating in lipid peroxidation and protein oxidation [52]. Lipid peroxidation can disrupt integrity of the phospholipid bilayer and inactivate membrane-bound receptors and enzymes, leading to increased cell permeability and death [53]. Moreover, ROS can diminish the cellular antioxidant capacity by promoting oxidation of the antioxidant enzymes [53]. In the present study, HFD-induced hyperlipidemia was associated with significant increase in hepatic lipid peroxidation and diminished GSH, SOD, CAT, and GPx. Thus, the suppression of hyperlipidemia and subsequent oxidative stress represents an effective approach to protect against CVDs and liver diseases. Several mechanisms of protection could be used by living cells to defend the oxidative processes, including antioxidant enzymes SOD, CAT, and GPx [54,55]. During oxidative stress, the body uses its defense mechanism to minimize the process of lipid peroxidation using the antioxidant enzymes. Herein, treatment of HFD rats with MEACL resulted in a significant increase in these endogenous enzymes. Also, reduced lipid peroxidation was observed by a significant decrease in MDA levels in liver homogenates and serum samples in a dose-dependent manner in different treated groups. Accordingly, *A. carambola* protected against oxidative damage in fluoride-induced liver and kidney toxicity [56] and chemically induced hepatocellular carcinoma [57] in rodents through inhibition of lipid peroxidation and restoration of antioxidants defenses.

Several studies have reported the presence of different phenolic compounds in *A. carambola*, such as epicatechin, gallic acid gallotanin forms, carambolaflavone, and proanthocyanidines, which are responsible for its antioxidant activity [58,59]. The present study revealed that apigenin is one of the flavonoids found in *A. carambola* extract. Apigenin protected against cellular oxidative stress by increasing the expression of antioxidant enzymes, including catalase and SOD [60,61]. Moreover, it was found to increase the expression of phase II enzyme-encoding genes by blocking the NADPH oxidase complex and their downstream target inflammatory genes and by enhancing the expression and nuclear translocation of nuclear factor erythroid 2-related factor 2 (Nrf2) [62,63].

To evaluate the effect of MEACL on lipid synthesis and excretion, TC and TG levels were measured in liver homogenate and feces samples. Cholesterol homeostasis is controlled by several mechanisms, such as cholesterol biosynthesis, absorption of dietary cholesterol, and cholesterol removal through biliary secretion [64]. For the mechanism involving biliary secretion, HDL-C plays a major role whereby it aids in the movement of excess cholesterol from the peripheral cells to the liver. The cholesterol is then secreted into bile as free sterol by ATP-binding cassette transporters or converted into bile acids with the help of rate-determining enzyme for bile acid biosynthesis, cholesterol 7α-hydroxylase (CYP7A1) [65]. Bile acids will then be secreted into the small intestine with bile, followed by reabsorption of 95% bile acids into the blood while the remaining 5% is secreted into feces [66]. The significant decrease in TC and TG levels in the liver and the increased excretion of TC in the feces at 500 and 1000 mg/kg MEACL shows that the extract helped in removing excess cholesterol. Bile acid excretion was evaluated by measuring fecal bile acids of all experimental groups at pretreatment and after five weeks of treatment with MEACL. The 500 and 1000 mg/kg doses of MEACL increased fecal cholesterol and bile acids.

MEACL showed antihyperlipidemic activity through multiple mechanisms, such as inhibition of HMG-Co A reductase and pancreatic lipase, reduction of the endogenous synthesis of TC and TG in the liver, and increase in bile acid secretion in the feces. In addition, the extract also possessed in vivo antioxidant activity, which may have potential effect in reducing cellular oxidation process. The antihyperlipidemic effect of MEACL in HFD-fed rats was comparable with another study by Visavadiya and Narasimhacharya who evaluated the hypolipidemic effect of sesame seed (*Sesamum indicum*) powder in male albino rats [67]. Administration of *S. indicum* powder to hypercholesterolemic rats resulted in a significant decline in plasma and hepatic total lipids, TC, and LDL-C levels. Furthermore, plasma HDL-C level was found to be increased. Moreover, the experimental animals showed increased fecal excretion of cholesterol, neutral sterol, and bile acids, along with increased hepatic HMG-CoA reductase activity. Additionally, sesame was found to improve the hepatic antioxidant status and reduce lipid peroxidation.

## 5. Conclusions

This study demonstrated that MEACL exhibited dose-dependent antihyperlipidemic effect, with the highest dose exerting the most potent effect in terms of reducing serum and tissue lipids in HFD-fed rats. It can be deduced that the beneficial properties of MEACL against hyperlipidemia may be mediated in part through multiple mechanisms, including inhibition of HMG-CoA reductase, increased secretion of cholesterol and bile acids in the feces, and reduction of the endogenous synthesis of TC and TG in the liver. In addition, the extract also increased the antioxidant defenses, which may have potential effect in reducing cellular oxidation process. Overall, this study suggests that MEACL has a potent antihyperlipidemic effect that could be further developed as a lipid-lowering agent.

## Figures and Tables

**Table 1:** HPLC mobile phase gradient program for apigenin analysis.

Time (min)	A (%)	B (%)
0.0	90.0	10.0
5.0	90.0	10.0
12.0	55.0	45.0
17.0	55.0	45.0
20.0	90.0	10.0

**Table 2:** Recovery precision and accuracy values for apigenin.

Amount Added (µg/mL)	Amount Measured (µg/mL)	Mean Recovery %	RSD %	CV
50	49.98	99.96	3.20	3.21
5	5.2	104.00	3.10	2.94
0.625	0.628	100.48	3.99	4.15

**Table 3:** Within-day and between-day precision and accuracy values for apigenin.

Concentration (µg/mL)	Within-day		Between-day	
	Accuracy (%)	Precision CV %	Accuracy (%)	Precision (CV%)
50	95.08	3.24	104.92	1.32
20	104.07	3.88	95.24	1.98
5	102.12	4.03	99.18	4.54
2.5	100.24	2.64	97.65	2.99
1.25	99.38	3.28	95.75	5.04
0.625	99.65	1.01	101.84	2.17

**Table 4:** The effect of MEACL on relative organ weight of HFD-fed rats.

Organ	Control	HFD	HFD + Atorvastatin	HFD + 250 mg/kg MEACL	HFD + 500 mg/kg MEACL	HFD + 1000 mg/kg MEACL
Liver	3.06 ± 0.10	4.78 ± 0.18	3.88 ± 0.24	3.91± 0.22	4.0 ± 0.13#	3.69 ± 0.15##
Brain	0.46 ± 0.02	0.38 ± 0.04	0.46 ± 0.01	0.59 ± 0.04	0.55 ± 0.05	0.48 ± 0.06
Heart	0.32 ± 0.02	0.35 ± 0.01	0.38 ± 0.01	0.40 ± 0.02	0.39 ± 0.01	0.35 ± 0.02
Lung	0.52 ± 0.02	0.48 ± 0.02	0.56 ± 0.02	0.57 ± 0.04	0.62 ± 0.04	0.60 ± 0.06
Thymus	0.12 ± 0.01	0.10 ± 0.01	0.14 ± 0.02	0.14 ± 0.03	0.11 ± 0.01	0.11 ± 0.02
R. kidney	0.28 ± 0.01	0.26 ± 0.01	0.36 ± 0.01	0.34 ± 0.02	0.34 ±0.01	0.33 ± 0. 02
L. kidney	0.29 ± 0.01	0.26 ± 0.01	0.34 ± 0.01	0.33 ± 0.01	0.34 ± 0.02	0.32 ± 0.02
R. adrenal	0.004 ± 0.0002	0.004 ± 0.0002	0.005 ± 0.0001	0.005 ± 0.0001	0.005 ± 0.0002	0.006 ± 0.0003
L. adrenal	0.005 ± 0.0002	0.005 ± 0.0003	0.005 ± 0.0002	0.006 ± 0.0004	0.005 ± 0.0001	0.005 ± 0.0002
Spleen	0.21 ± 0.01	0.19 ± 0.02	0.24 ± 0.01	0.27 ± 0.03	0.26 ± 0.02	0.21 ± 0.01
Stomach	1.28 ± 0.09	1.40 ± 0.10	0.63 ± 0.06	1.06 ± 0.16	1.19 ± 0.16	1.18 ± 0.14
Adipose tissue	1.54 ± 0.20	1.50 ± 0.14	1.59 ± 0.23	1.54 ± 0.201	1.55 ± 0.27	0.89 ± 0.08#
Gut	5.42 ± 0.23	5.00 ± 0.13	4.58 ± 0.16	5.08 ± 0.35	5.73 ± 0.36	5.56 ± 0.44
R. tests	0.38 ± 0.03	0.35 ± 0.02	0.50 ± 0.01	0.50 ±0.03	0.54 ± 0.02	0.53 ± 0.03
L. tests	0.43 ± 0.04	0.35 ± 0.02	0.51 ± 0.01	0.51 ± 0.02	0.55 ± 0.02	0.54 ± 0.03

Abbreviations: L, left, R, right. Values are expressed as mean ± SEM, *n* = 6. # *p* < 0.05, ## *p* < 0.01 compared to hyperlipidemic control.

## Supplementary Materials

Supplementary materials can be found at https://www.mdpi.com/2227-9059/7/3/72/s1. Table S1: LOD, LOQ and linearity of standard curves for apigenin, Table S2: Contents of apigenin in methanolic extract (5 mg/mL) of *Averrhoa carambola* leaf, Figure S1: UV-vis spectrum of apigenin, Figure S2: A typical calibration curve of apigenin (50–0.625 µg/mL), Figure S3: HPLC chromatogram for apigenin standard at 0.1 µg/ml methanol.
