# Supplementary material for: Antihyperlipidemic and Antioxidant Effects of Averrhoa Carambola Extract in High-Fat Diet-Fed Rats
Source: Biomedicines. 2019 Sep 16;7(3):72. doi: 10.3390/biomedicines7030072 (PMC6784245; doi:10.3390/biomedicines7030072)
Supplement: Supplementary file 1 [file biomedicines-07-00072-s001.pdf]

Supplementary material

Antihyperlipidemic and antioxidant effects of *Averrhoa carambola* extract in high fat diet-fed rats

Saleem H. Aladaileh[1], Sultan A. M. Saghir [1, 2,*], Kisantini Murugesu[2], Amirin Sadikun[3], Ashfaq Ahmad[4], Gurjeet Kaur[5], Ayman M. Mahmoud[6] and Vikneswaran Murugaiya[2]

Table S1. LOD, LOQ and linearity of standard curves for apigenin.

Retention time/min.	Linear range (μg/mL)	LOD (μg/mL)	LOQ (μg/mL)	R^2
13.56	0.625–50.00	0.15	0.63	0.999

Table S2. Contents of apigenin in methanolic extract (5 mg/mL) of *Averrhoa carambola* leaf.

Apigenin	Calibration curve	Amount (μg/mg)
Methanolic extract of *A. carambola* leaf	y =42.882x − 26.83	4.83

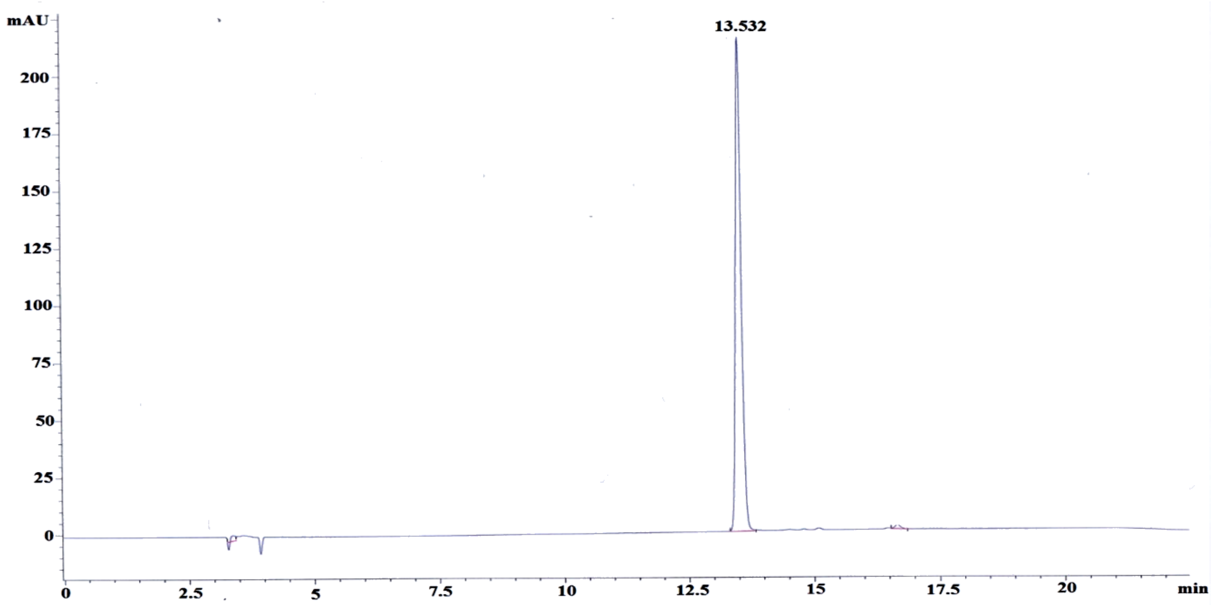

Figure S1. HPLC chromatogram for apigenin standard at 0.1 μg/ml methanol.

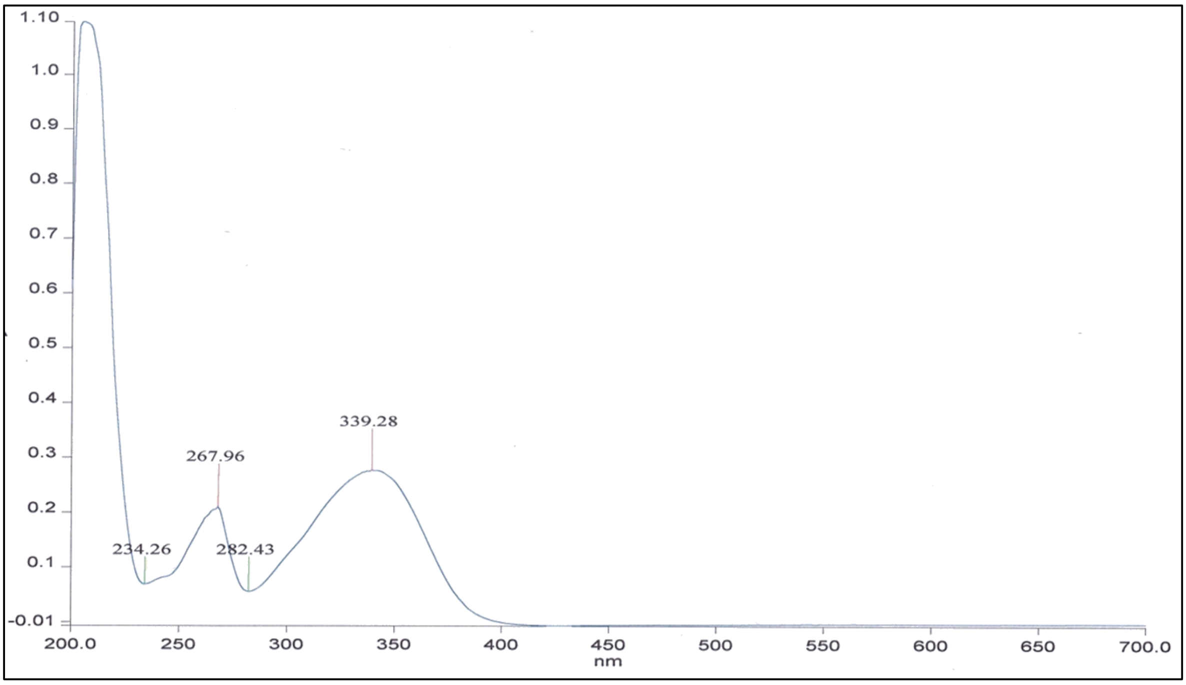

Figure S2. UV-vis spectrum of apigenin.

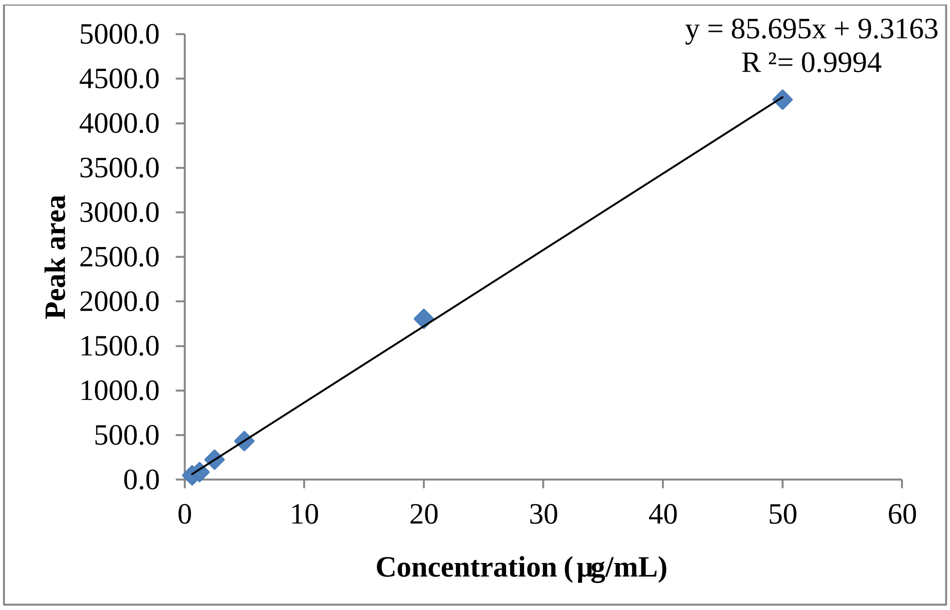

Figure S3. A typical calibration curve of apigenin (50–0.625 µg/mL).
